# Association of IL-17F rs2397084 (E126G), rs11465553 (V155I) and rs763780 (H161R) variants with rheumatoid arthritis and their effects on the stability of protein

**DOI:** 10.1371/journal.pone.0285874

**Published:** 2023-09-26

**Authors:** Yasir Ali, Masood Kausar, Mazhar Farooq, Nadia Farooqi, Zia Ul Islam, Suleman Khan, Aisha Aman, Naveed Khan, Atif Kamil, Fazal Jalil

**Affiliations:** 1 Department of Biotechnology, Abdul Wali Khan University Mardan, Khyber Pakhtunkhwa, Pakistan; 2 School of Biomedical Sciences, Chinese University of Hong Kong, New Territories, Hong Kong; 3 Consultant Rheumatologists, Lady Reading Hospital-MTI Peshawar, Khyber Pakhtunkhwa, Pakistan; Nazarbayev University School of Medicine, PAKISTAN

## Abstract

Interleukin-17F (IL-17F), considered a pro-inflammatory cytokine, has been shown to contribute to skeletal tissue degradation and hence chronic inflammation in rheumatoid arthritis (RA). In this study we utilized bioinformatics tools to analyze the effect of three exonic SNPs (rs2397084, rs11465553, and rs763780) on the structure and function of the IL-17F gene, and evaluated their association with RA in Pakistani patients. The predicted deleterious and damaging effects of identified genetic variants were assessed through the utilization of multiple bioinformatics tools including PROVEAN, SNP&GO, SIFT, and PolyPhen2. Structural and functional effects of these variants on protein structures were evaluated through the use of additional tools such as I-Mutant, MutPred, and ConSurf. Three-dimensional (3D) models of both the wild-type and mutant proteins were constructed through the utilization of I-TASSER software, with subsequent structural comparisons between the models conducted through the use of the TM-align score. A total of 500 individuals, 250 cases and 250 controls, were genotyped through Tri-ARMS-PCR method and the resultant data was statistically analyzed using various inheritance models. Our bioinformatics analysis showed significant structural differences for wild type and mutant protein (TM-scores and RMSD values were 0.85934 and 2.34 for rs2397084 (E126G), 0.87388 and 2.49 for rs11465553 (V155I), and 0.86572 and 0.86572 for rs763780 (H161R) with decrease stability for the later. Overall, these tools enabled us to predict that these variants are crucial in causing disease phenotypes. We further tested each of these single nucleotide variants for their association with RA. Our analysis revealed a strong positive association between the genetic variant rs763780 and the risk of developing rheumatoid arthritis (RA) at both the genotypic and allelic levels. The genotypic association was statistically significant[*χ*^2^ = 111.8; P value <0.0001], as was the allelic level [OR 3.444 (2.539–4.672); P value 0.0008]. These findings suggest that the presence of this genetic variant may increase the susceptibility to RA. Similarly, we observed a significant distribution of the genetic variant rs11465553 at the genotypic level [***χ***^**2**^ = 25.24; P value = 0.0001]. However, this variant did not show a significant association with RA at the allelic level [OR = 1.194 (0.930–1.531); P value = 0.183]. However, the distribution of variant rs2397084 was more or less random across our sample with no significant association either at genotypic and or allelic level. Put together, our association study and in silico prediction of decreasing of IL17-F protein stabilty confirmed that two SNPs, rs11465553 and rs763780 are crucial to the suscetibility of and showed that these RA in Pakistani patients.

## Introduction

Rheumatoid Arthritis is an inflammatory autoimmune disorder which mainly affects the joints, resulting in high levels of rheumatoid factor (RF) and anti-citrullinated protein antibody (ACPA) [1]. RA is a devastating disease, causing bone and cartilage deformities, and systemic injuries, if left untreated and unmanaged [2]. Its complications can be mild joints swelling or severe polyarthritis related with erosion of bones or cartilages [3]. The prevalence of RA remains relatively consistent across various populations worldwide, with a global incidence of 0.5 to 1.0%. [4]. Although its pathogenesis is still unclear, many genetic, environmental, hormonal, and infectious factors have significant roles in RA [5]. Approximately 50–60% of RA cases can be attributed to genetic factors [6]. The genetic factors include polymorphisms in cytokine receptors as well as in other functional pathways genes. However, several association studies and meta-analyses have linked about 150 genes/loci with RA in various populations [7–11]. Genetic variations in Th17 cytokines could affect the transcriptional regulation of these genes, which in response; increase the susceptibility of patients to diseases. For example, the polymorphisms in Th17 regimens are related with different complex diseases, including RA, Crohn’s disease, multiple sclerosis, and juvenile idiopathic arthritis [12]. Nearly 20 genes are known to be closely associated with the development and activity of Th17 cells [13].

The IL-17 family of proinflammatory cytokines is made up of six distinct members: IL-17A, IL-17B, IL-17C, IL-17D, IL-17E, and IL-17F [14]. Despite the co-localization of the genes encoding IL-17A and IL-17F on the chromosome 6p12, other members of the IL-17 family are distributed across different chromosomes [15–17]. The expression of IL-17A and IL-17F stimulate the generation of additional cytokines, chemokines, and antimicrobial peptides, leading to the degradation of skeletal tissues and chronic inflammation in affected individuals [15]. The expression of both IL-17A and IL-17F is detectable within synovial tissue, and their presence has been implicated in the pathogenesis of rheumatoid arthritis (R [18]. Based on in vitro evidence demonstrated that IL-17F may be involved in the regulation of angiogenesis, as well as the production of specific cytokines from both endothelial cells (including CXCL1, ICAM1, IL-6, and IL-8) and epithelial cells (such as G-CSF) [19–22].

Recent evidence has revealed that the H161R variant functions as a natural antagonist to wild-type IL17F. This is achieved through its ability to bind to the IL17F receptor without inducing downstream signaling pathways, effectively blocking the induction of IL8 [23, 24]. Several inflammatory diseases, including rheumatoid arthritis, inflammatory bowel disease, asthma, Graves’ disease, ulcerative colitis, and cancer, have identified IL-17F as a promising candidate gene. These findings highlight the potential involvement of IL-17F in the pathogenesis of these conditions and suggest that targeting this cytokine may hold therapeutic promise [25–32]. Numerous studies have demonstrated a clear association between levels of IL-17 in both serum and synovial fluid and markers of inflammation, including rheumatoid factor (RF), C-reactive protein (CRP), erythrocyte sedimentation rate (ESR), and the Disease Activity Score 28 (DAS-28) in patients with rheumatoid arthritis (RA) [33].

Being an important component of inflammatory pathway, the single nucleotide variants of *IL-17F* have been subjected to association studies and the link with RA has been reported for rs2397084, rs11465553 and rs763780 variants in various populations [14–19]. Each of these variants lies in exonic regions of *IL-17F* gene and result in non-synonymous changes in the encoded protein.

The epigenome encompasses a variety of mechanisms that modulate gene expression patterns and phenotypic outcomes in response to environmental factors, such as nutrition, pathogens, and climate. These mechanisms include DNA methylation, chromatin remodeling, histone tail modifications, microRNAs, and long noncoding RNAs. The complex interplay between the epigenome and environmental cues highlights the crucial role of epigenetic regulation in shaping biological processes and underscores its potential as a therapeutic target for a broad range of diseases. [20, 21]. Environment, genome and epigenome might be involved in multi-level interaction [22]. Additionally, epigenome variation appears to have an impact on health and productivity [23–25]. In eukaryotes, gene expression is regulated through complex, temporal, and multidimensional mechanisms [26]. In each type of tissue, only a small fraction of the genome is expressed, and gene expression varies with developmental stage; therefore, eukaryotes express genes differently based on their tissues [27, 28]. The expression of a gene is influenced by the amount of its gene products in the tissue where it is expressed, as well as in other tissues that contribute to the formation of the gene product [29]. The analysis of genes and proteins associated with diseases and important traits entails studying at the cellular or chromosomal level 1 [30].

In this study we applied various bioinformatics tools to determine the effect of, rs11465553 (V155I), rs2397084 (H161R) and rs763780 (E126G) on the structure, stability and hence function of IL-17 F protein. Apart from *in silico* analysis, we also performed a case control study to determine the association between these three SNPs and RA in Pakistani population.

## Methodology

**Fig 1:**
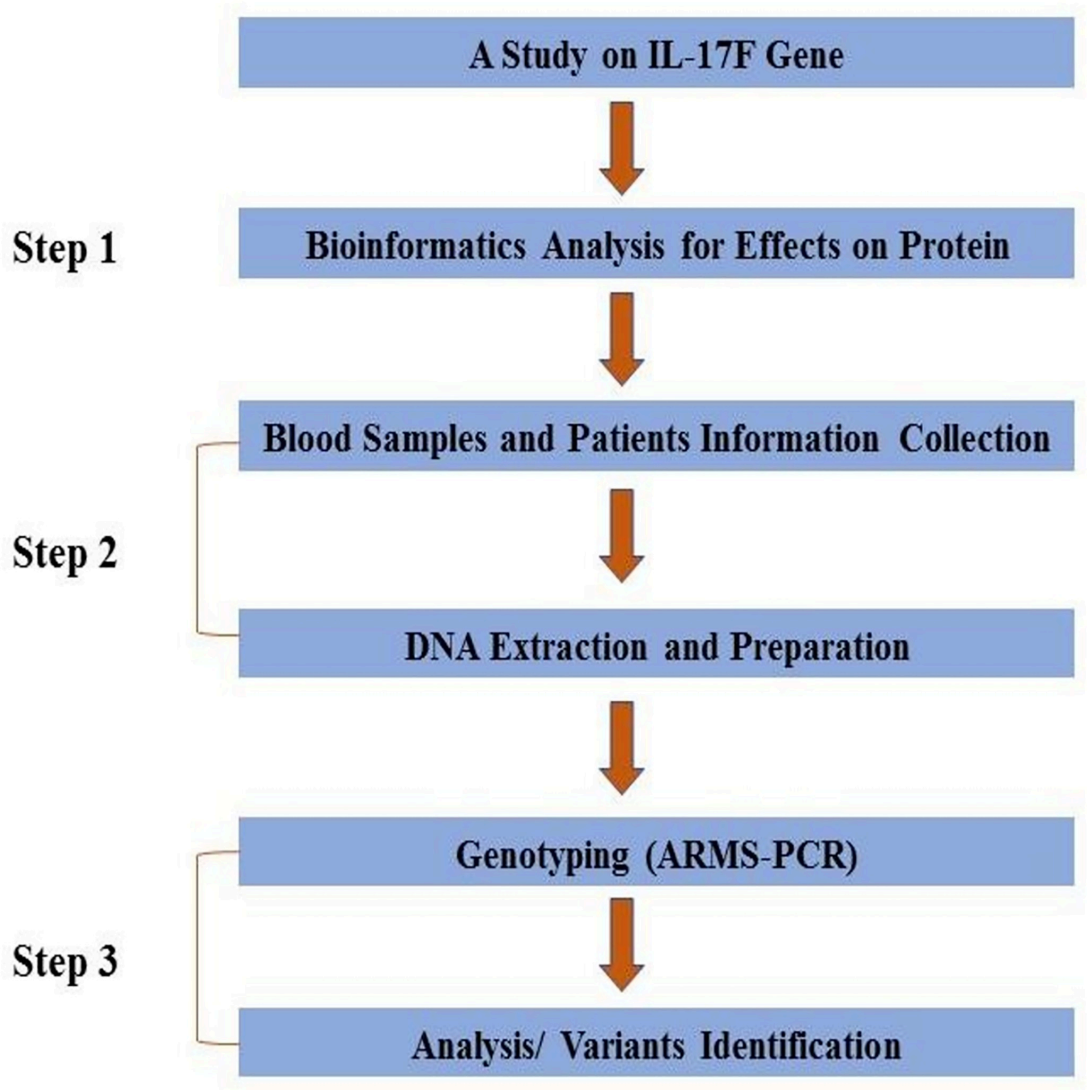
Workflow of the study.

A workflow for the complete methodology is given in (Fig 1).

### Bioinformatics analysis

Different bioinformatics tools, including PhD-SNP (Predictor of human Deleterious SNP) (https://snps.biofold.org/phd-snp/phd-snp.html) [31], SNPs&GO (https://snps.biofold.org/snps-and-go/snps-and-go.html) [32], SIFT (Sorting Intolerant from Tolerant) (https://sift.bii.a-star.edu.sg/) [33], PROVEAN (Protein Variation Effect Analyzer) (http://provean.jcvi.org/seq_submit.php) [34], and PolyPhen2 (Polymorphism Phenotyping 2) (http://genetics.bwh.harvard.edu/pph2/) [35] were applied to evaluate deleterious effect of these variants. Structural and functional effect of the variants was predicted by MutPred (http://mutpred.mutdb.org/) [36] and their effects on the stability of the targeted protein were analyzed using I-Mutant 2.0 (http://folding.biofold.org/i-mutant/i-mutant2.0.html) [37]. Furthermore, the ConSurf server (http://consurf.tau.ac.il/2016/) helped to predict the evolutionary conserved residues [38]. For 3D modeling of these proteins, the Protein Data Bank (PDB) file was produced by I-TASSER (https://seq2fun.dcmb.med.umich.edu//I-TASSER/) [39]. Chimera 1.11 was used for the molecular properties and visualization of resultant proteins [40]. We compared the structures of wild type and mutant proteins using TM-align (https://seq2fun.dcmb.med.umich.edu//TM-align/example/873772.html), a computational tool that calculates TM scores and RMSD values. This helped us understand how the mutation impacted the protein’s shape and function. [41].

### Genetic analysis

For genetic analysis, 250 seropositive RA patients (187 females, 63 males) with the mean age of 43.5 (±14.5) years were recruited from Department of Rheumatology, Lady Reading Hospital (LRH) Peshawar, Pakistan. The patients’ diagnoses were made by experienced rheumatologists who followed the rigorous guidelines set out by the American College of Rheumatology (ACR) [42]. Each patient was examined for the number of joints involved, the extra-articular manifestations as well as for the clinical features, such as rheumatoid factor (RF), erythrocyte sedimentation rate (ESR) and ACPA. Similarly, a control group of 250 healthy individuals (181 female, 69 males) was included for comparison. The mean age of the control group was 42 (±12.6) years with a standard deviation. Individuals with any other autoimmune complex disease or family history of such illnesses were excluded. Prior to the study, written informed consent was obtained from all participants, and the study was conducted in accordance with ethical guidelines set by both the Abdul Wali Khan University Mardan and LRH Peshawar in Pakistan. The clinical characteristics of the rheumatoid arthritis (RA) patients are detailed in Table 1.

**Table 1:** Characteristics of both rheumatoid arthritis patients and healthy controls.

Variable	Cases (n = 250)	Controls (n = 250)	*P* value
**Gender (male/female)**	63/187	69/181	0.6121
**Age in years Mean (±SD)**	43.5 (±14.5)	42 (±12.6)	0.217
**Disease duration in years Mean (±SD)**	4.1 (±3.7)	-	-
**Sero-positive antibody Mean (±SD)**	100% (RF positive)	-	-
**ESR Mean (±SD)**	40.60 (±15.8)	-	-

Blood sample (5 mL) was taken from each participant and DNA was isolated via phenol-chloroform method [43]. Three *IL-17F* gene SNPs rs2397084, rs11465553, and rs763780 were genotyped using Amplification Refractory Mutation System-Polymerase Chain Reaction (ARMS-PCR)with allele-specific primers (two forward and one reverse) and details of the nucleotide has been provided in Table 2. The primer sets included the following primers; 1) for *IL-17F-*rs2397084 (T/C), F1: 5’-TCCGGACGACCAGGGTCC-3’; F2: 5’-CTCCGGACGACCAGGGTCT R: 5’-CCAGGCTGTGTGGCTCCAGAA-3’. 2) for *IL-17F*-rs11465553 (A/G), F1: 5’-TGACTGTTGGCTGCACCTGCA-3’; F2: 5’-ACTGTTGGCTGCACCTGCG-3’; R: 5’-CTGTTTCCATCCGTGCAGGTC-3’; and 3) for *IL-17F*-rs763780 (C/T), F1: 5’-ATATGCACCTCTTACTGCACAC-3’; F2: 5’-GATATGCACCTCTTACTGCACAT-3’; R: 5’-TACCCCTCGGAAGTTGTACAG-3’. To amplify our DNA samples and study the IL-17F gene SNPs, we used a carefully crafted set of PCR conditions. First, we began with an initial denaturation step at 94°C for 5 minutes, to help prepare the DNA strands for amplification. Next, we embarked on a series of 35 cycles, each of which included denaturation at 94°C for 30 seconds, annealing at a temperature range of 57–60°C for 30 seconds, and extension at 72°C for 1 minute. These cycles were designed to efficiently amplify the specific regions of interest in the DNA samples. Finally, we performed a final extension step at 72°C for 7 minutes to ensure that all of the amplified DNA fragments were completely synthesized. With these carefully controlled PCR conditions, we were able to generate high-quality DNA amplification products for our research. We used a 2% agarose gel to separate the amplified products and looked for distinct band patterns. By comparing these patterns to known standards, we were able to accurately determine the genotype calls for each sample.

**Table 2:** Details of amino acid changes in the *IL17-F* gene.

Gene	SNP ID	Reference Allele	Alter Allele	Amino acid Changes
**IL-17F**	rs2397084	T	C	Glu126Gly (E126G)
	rs11465553	G	A	Val155Ile (V155I)
	rs763780	C	T	His161Arg (H161R)

The data was subjected to quality controls check using Hardy-Weinberg Equilibrium (HWE), and association of *IL-17F* rs2397084, rs11465553, and rs763780 with RA was tested using the statistical models. We performed statistical analyses using both the Chi-square (χ2) and Fisher’s exact tests, with a confidence interval of 95% (95% CI). The Chi-square test was utilized for larger sample sizes to compare observed and expected frequencies, while Fisher’s exact test was used for smaller sample sizes to calculate the probability of obtaining a frequency distribution under the assumption of independence. We only considered a P-value less than 0.05 to be statistically significant in all of our analyses.

## Results

### Bioinformatics analysis

**Fig 2:**
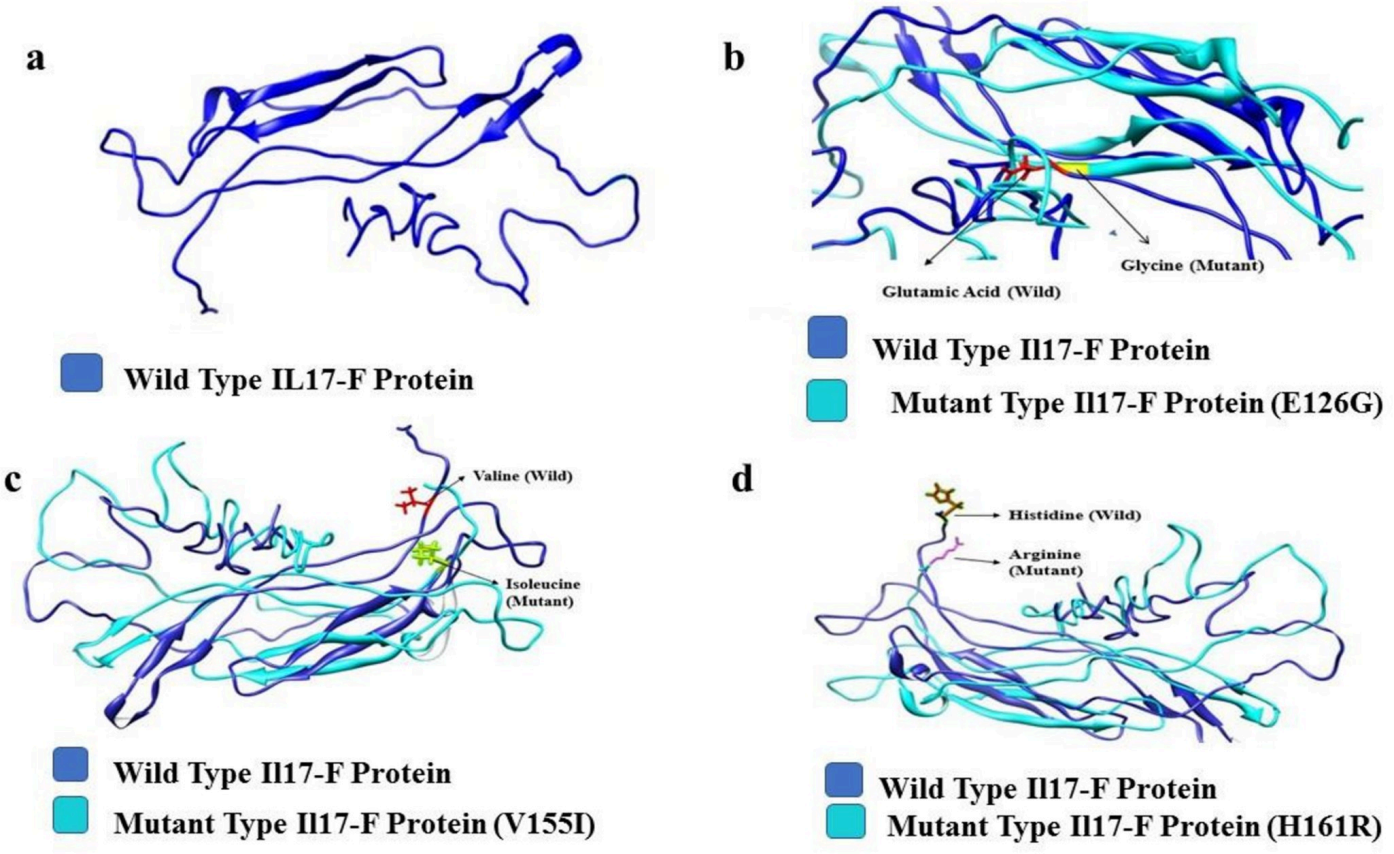
3-D structural models visualized in Chimera 1.11 (a). wild type IL-17F protein (b). IL-17F superimposed with E126G mutant (c). IL-17F superimposed with V155I mutant (d). IL-17F superimposed with H161R mutant.

The results of bioinformatics analyses are listed in Table 3. In PhD-SNP and SNPs&GO tools, the SNPs with a prediction score >0.5 were evaluated deleterious. The scores for each variant (E126G, V155I, and H161R) were less than 0.5, which were evaluated to be neutral. In SIFT the cut-off value for tolerance index (TI) was set to 0.05. The variant V155I (0.12) was found to be tolerated, while the E126G (0.02) and H161R (0.05) were evaluated as deleterious through SIFT. The PROVEAN (threshold value -2.5) predicted V155I (-0.926) as neutral and E126G (-5.786) and H161R (-2.620) variants as deleterious. PolyPhen2 (score range 0–1) predicted V155I (1) and E126G (0.999) as probably damaging and H161R (0.048) as a benign variant. Furthermore, I-Mutant predicted that all the three variants decreased the stability of IL-17F protein [E126G (RI = 9); V155I (RI = 4); H161R (RI = 5)]. MutPred tool predicted structural and functional consequences including gain of intrinsic disorder, loss of allosteric site, creation of glycosylation and catalytic site, and altered membrane protein etc. Only H161R showed loss of strand, altered transmembrane protein, and altered protein stability. ConSurf predicted that the variants E126G and V155I were highly conserved, exposed and functional residues while H161R was highly conserved and exposed but not functionally active residue. The 3-D proteins structures were produced using I-TASSER and were subjected to TM-align analysis. Thus, significant structural changes were determined through TM-scores and RMSD values of the variants. TM-scores and RMSD values were 0.85934 and 2.34 for E126G, 0.87388 and 2.49 for V155I, and 0.86572 and 0.86572 for H161R, respectively. Finally, protein structures were viewed and characterized through Chimera 1.11and are shown in (Fig 2).

**Table 3:** Bioinformatic analyses of *IL-17F* gene.

Bioinformatics tools	rs2397084 (E126G)		rs11465553 (V155I)	rs763780 (H161R)
**PHD SNP (Threshold 0.5)**	Prediction	Neutral	Neutral	Neutral
	Score	0.145	0.381	0.138
**SNP& GO (Threshold 0.5)**	Prediction	Neutral	Neutral	Neutral
	Score	0.145	0.159	0.050
**SIFT**	Prediction	Not-tolerated	Tolerated	Not-tolerated
	Score	0.02	0.12	0.05
**PROVEAN**	Prediction	Disease	Neutral	Disease
	Score	-5.786	-0.926	-2.620
**POLYPHEN-2**	Prediction	Probably Damaging	Probably Damaging	Benign
	Score	1	0.999	0.048
**I-Mutant**	Prediction	Decrease	Decrease	Decrease
	Score	9	4	5
**ConSurf**	Prediction	Highly Conserved and Exposed (F)	Highly Conserved and Exposed (F)	Highly Conserved and Exposed
	Conservation score	9	8	5
**MutPred**	Top Features	-	-	LOSS Of Strand (P = 0.05) Altered Transmembrane protein (P = 1.3e-03) Altered Stability (P = 0.03)

0.0< TM-score < 0.30, random structural similarity 0±0.3 and 0.5 < TM-score < 1.00, in about the same fold 0.5±1.

### Association analysis

**Fig 3:**
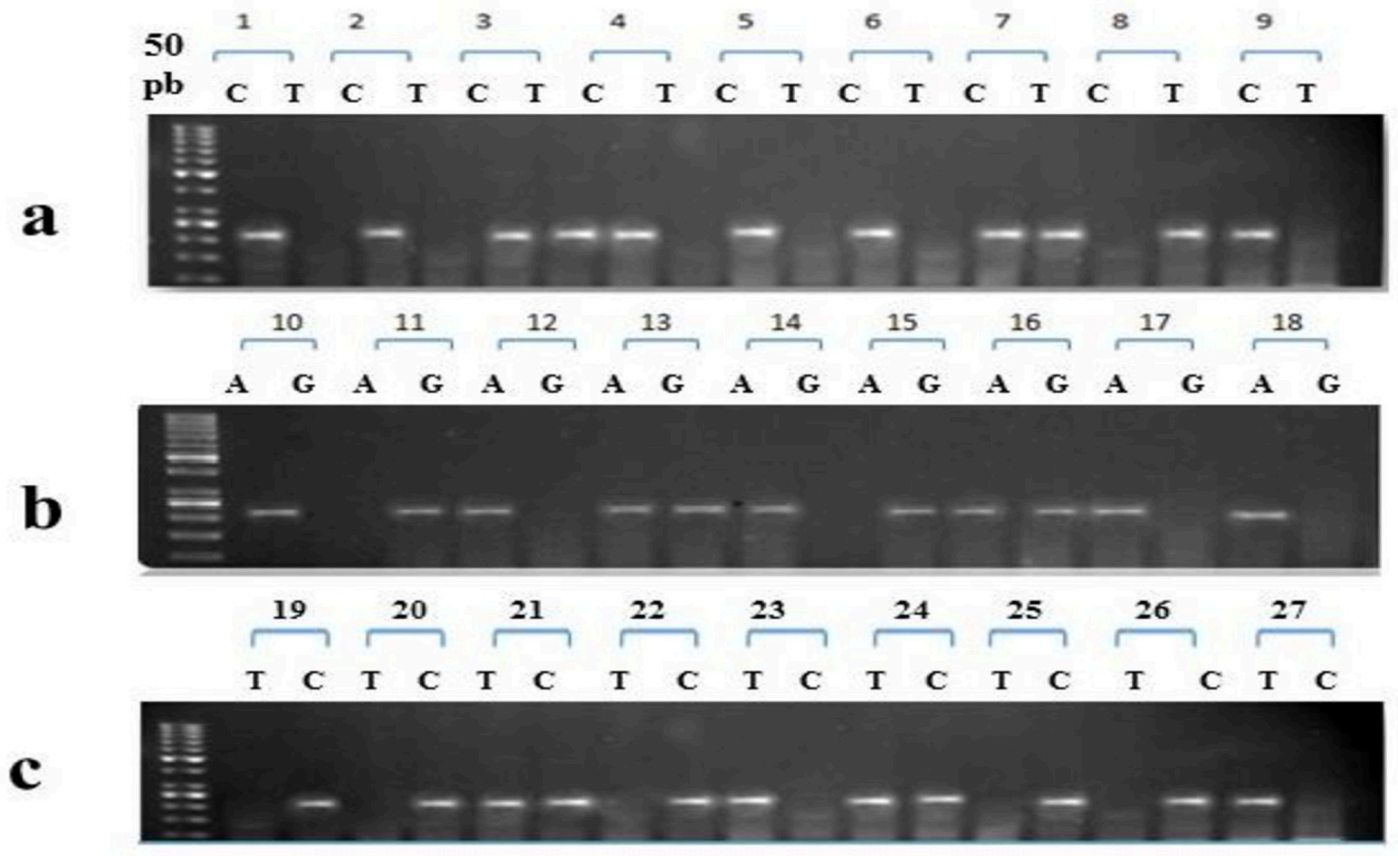
Tri-Primer ARMS-PCR Gel Pattern of IL17-F Gene, a) IL-17F rs2397084 Genotypes, b) IL-17F *rs*11465553 Genotypes, and c) IL-17F rs763780 Genotypes.

The findings of association analyses are given in Table 4 and gel electrophoresis pattern are shown in (Fig 3). An insignificant distribution of rs2397084 was observed at both the genotypic [*χ*^2^ = 3.202; P value 0.201] and allelic levels [OR 0.858 (0.668–1.101); P value 0.253]. Whereas, in case of rs11465553 a significant distribution of genotypes was observed in co-dominant [***χ***^**2**^ = 25.24; P value 0.0001] and homozygous models [OR 0.415 (0.268–0.641); P value 0.0001], but the distribution of its genotypes was insignificant at allelic level [OR 1.194(0.930–1.531); P value 0.183]. Similarly, the distribution of rs763780 in cases and controls was significant at genotypic [*χ*^2^ = 111.8; P value 0.0001] as well as allelic level [OR 3.444(2.539–4.672); P value 0.0008].

**Table 4:** Statistical models used in association analysis.

Genes	Models	Genotype	Cases 250	Controls 250	OR 95% Cl	*χ*^2^ -value	P-value
***IL17F* rs2397084**	Co-		n (% age)	n (% age)			
	Dominant	CC	32 (13%)	35 (14%)	-	3.202	0.201
	Model	CT	160 (64%)	173 (70%)			
		TT	58 (23%)	42 (16%)			
	Homozygous	TT	58 (23%)	42 (16%)	1.496		
	Dominant	CT + CC	192 (77%)	208 (84%)	(0.960–2.330)	-	0.093
	Homozygous	CC	32 (13%)	35 (14%)	0.901		
	Recessive	CT + TT	218 (87%)	215 (86%)	(0.538–1.509)	-	0.793
	Heterozygou	CT	160 (64%)	173 (70%)	0.791		
	s	CC+TT	90 (36%)	77 (30%)	(0.549–1.155)	-	0.255
	Additive	C	224 (44.8%)	243 (48.6%)	0.858		
		T	276 (45.2%)	257 (51.4%)	(0.668–1.101)		0.253
***IL17F* rs11465553**	Co-	AA	32 (13%)	48 (18%)			
	Dominant	AG	179 (72%)	125 (50%)	-	25.24	0.0001
	Model	GG	39 (16%)	77 (32%)			
	Homozygous	GG	39 (16%)	77 (32%)	0.415		
	Dominant	AG +AA	211 (84%)	173 (68%)	(0.268–0.641)	—	0.0001
	Homozygous	AA	32 (13%)	48 (18%)	0.617		
	Recessive	GG +AG	218 (87%)	202 (82%)	(0.379–1.005)	—	0.066
	Heterozygou	AG	179 (72%)	125(50%)	1.19		
	s	AA+GG	71 (28%)	125 (50%)	(0.790–1.791)	**—**	0.466
	Additive	A	243 (48.6%)	221 (44.2%)	1.194		
		G	257 (51.4%)	279 (54.8%)	(0.930–1.531)	**—**	0.183
***IL17F* Irs763780**	Co-	TT	79 (31.6%)	192 (76.8%)			
	Dominant	CC	18 (7.2%)	17 (6.8%)	-	111.8	<0.0001
	Model	CT	153 (61.2%)	41 (16.4%)			
	Homozygous	TT	79 (39%.76)	192(28.23%)	0.139		
	Dominant	CT + CC	171(60.24%)	58(61.67%)	(0.093–0.027)	-	0.001
	Homozygous	CC	18 (16.20%)	17 (21.08%)	1.063		
	Recessive	CT + TT	232 (83.80%)	233 (78.92%)	(0.534–2.115)	-	1.00
	Heterozygou	CT	153 (61.2%)	41 (16.4%)	4.193		
	s	CC+TT	97 38.8%)	109(83.6%)	(2.70–6.51)	-	0.0001
	Additive	C	189(37.8%)	75 (15%)	3.444		
		T	311 (62.2%)	425 (85%)	(2.539–4.672)		0.0001

## Discussion

IL-17 plays a crucial role in rheumatoid arthritis (RA) by promoting inflammation and angiogenesis. In the early stages of RA, IL-17 activates fibroblast-like synoviocytes (FLS) to produce vascular endothelial growth factor (VEGF), which leads to increased angiogenesis and inflammation in the joint [44]. IL-17 also boosts the production of inflammatory mediators such as IL-6, IL-8, prostaglandin E2 (PGE2), and granulocyte colony-stimulating factor (G-CSF) from synovial fibroblasts [45–47]. Besides RA, many studies have elucidated relationship of *IL-17F* rs2397084 (E126G) and rs763780 (H161R) polymorphisms to osteoarthritis [48], IBD and UC [49]. Similarly, The IL-17F H161R genetic variation has also been studied in relation to various other disorders., including functional dyspepsia (FD), BD, chronic fatigue syndrome (CFS) or gastric cancer [50] FD and *H*. *pylori*-infected patients [51].

The in-silico and computational methods have identified several variants that significantly affect the structure and function of certain proteins [52–55]. Therefore, we utilized multiple in-silico tools to assess the deleterious impacts of the variants on the structure and function of the IL17F protein. Bioinformatics tools predicted deleterious effect of variants and the findings were cross-checked with CADD, REVEL, Mutation Assessors and MetalR in Ensemble genome browser 96 (accessed: 25th January, 2022). When a variant receives a CADD score of 30, it falls within the top 0.1% of the most damaging single nucleotide polymorphisms (SNPs) known to science. Similarly, a CADD score of 20 signifies that the variant is among the top 1% of the most harmful SNP variants found throughout the entirety of the human genome. The CADD score of E126G variant was 27 followed by V155I and H161R that were 23 and 21, respectively. MutPred1.2 server predicted that E126G variant had highest P-value of 0.677, followed by V155I and H161R (0.252 and 0.088, respectively). These results suggested that these variants may structurally and functionally affect IL17F protein. Furthermore, I-Mutant predicted that these variants decreased the protein stability, as listed in Table 3. To ensure the reliability of our findings, we double-checked the results from I-Mutant by comparing them with those generated by the CUPSAT server (http://cupsat.tu-bs.de/), which ultimately provided further support for our initail findings. ConSurf analysis facilitated the assessment of protein evolutionary conservation, with the identification of the most highly conserved amino acids providing key insights into their fundamental role in protein structure and function [38]. Furthermore, the highly conserved residues are very important for protein-protein interactions. According to Miller and Kumar, highly conserved nsSNPs are most damaging ones [33].

In this study, the variants E126G and V155I were identified within a region of high evolutionary conservation, and were predicted to be critical for protein function., while H161R was functionally inactive. The protein structure was modelled using I-TASSER with protein FASTA sequences. We utilized the RAMPAGE server to perform a Ramachandran Plot Analysis and compute RAMPAGE values for the modeled protein structures [56]. Protein structures with RAMPAGE values > 80% were considered as a better structure [57]. The wild type IL17-F protein achieved a RAMPAGE value of 84.8% (favored), 12.5% (allowed), and (2.7%) outlier. Similarly, for the variant E126G; 83.4% (favored), 11.5 (allowed), 5.1 (outlier), and for V155I; 81.2% (favored), 11.6% (allowed), 7.2% (outlier), and for H161R 85.4% (favored), 11.5% (allowed), 3.1% (outlier).

In this study, we analyzed three variants (rs763780, rs11465553 and rs2397084) of *IL-17F* genes for their association with RA and found significant association for two of them (rs763780 and rs11465553) in Pakistani population. Contrary to our findings, a case-control study on Polish population reported no association of IL-17F rs763780 and rs2397084 with RA. However, a significant association was observed, for rs763780, upon stratification of individuals, based on joints tenderness, HAQ score or DAS-28-CRP level. Furthermore, rs2397084 was associated with longer disease activity in RA cases [14]. Similarly, another Polish study reported IL-17F rs763780 as risk factor of RA [15]. A group of investigators, genotyped IL-17F (rs763780, rs11465553, rs2397084) using RFLP method and established an insignificant distribution in Turkish population [16]. There were different observations even in the same population, when the literature was reviewed. A comparatively larger case-controls study on individuals of Polish decent (422 RA cases and 337 control) determined an insignificant association of IL-17F (rs763780, rs11465553, rs2397084) through TaqMan assays [17]. Marwa et al. performed a case-control study in Tunisian population using RFLP method and showed a significant association of IL-17F rs2397084 and rs763780 with RA [19]. In a meta-analysis of 7,474 patients and 10,628 controls, obtained from 25 different studies, established that IL-17F rs763780 was significantly involved with increasing the RA risk [18]. To our knowledge, genetic factors have rarely been studied in Pakistanis. Few studies have replicated the known SNPs/variants in Pakistani RA patients [11, 58–61]. Recently, IL-17F gene was sequenced in 50 RA cases and 50 controls of Pakistani origin and established a significant association of rs763780 [62]. Furthermore, there is no newly established and authentic data available on the prevalence of RA in Pakistani population. However, the trend in disease progression with respect to gender differences have been shown [63], with the prevalence rate of 0.14% [64].

There is a limitation of the study. We did not perform haplotype analyses of the three SNPs and the information of linkage disequilibrium is not available. Although our study provides valuable insights, it is important to note that ethnic variability may exist, highlighting the need for large-scale, multi-ethnic population studies to fully elucidate the pathogenic mechanisms and evolutionary background of the genetic factors implicated.

## Conclusions

It is concluded that *IL-17F* rs763780 (H161R) and rs11465553 (V155I) are the risk factors for RA in Pakistani patients. Furthermore, these are important coding variants, encoding highly conserved amino acids. Thus, these variants decrease the stability of IL-17F protein, thereby causing structural changes. Thus, on the basis of these findings IL-17F can be considered to be one of the potential therapeutic targets.
